# Low m6A modification-mediated upregulation of PLAC8 promotes trophoblast cell invasion and migration in preeclampsia

**DOI:** 10.1186/s40001-023-01442-7

**Published:** 2023-10-27

**Authors:** Yajuan Zhang, Xiaoguang Guo, Zhimin Chen, Ruixia Guo

**Affiliations:** 1 Department of Obstetrics and Gynecology, The First Affiliated Hospital of Zhengzhou University, 1st Jianshe East Road, Zhengzhou, Henan 450000 China; 2 Department of Anesthesiology, Pain and Perioperative Medicine, The First Affiliated Hospital of Zhengzhou University, Zhengzhou, Henan 450000 China

**Keywords:** Preeclampsia, m6A, PLAC8, Invasion, Migration, Trophoblasts

## Abstract

**Background:**

The main symptoms of preeclampsia (PE), a specific ailment that develops during pregnancy, are proteinuria and hypertension. The pathological root of the onset and progression of PE is widely regarded as abnormal placental trophoblast cell function. This study aimed to look into the character and mechanism of Placenta-specific 8 (PLAC8) in trophoblast cell invasion and migration.

**Methods:**

Expressions of PLAC8 and AlkB homologue 5 (ALKBH5) were examined by western blot and quantitative real-time PCR. The m6A level of PLAC8 mRNA was detected by methylated RNA Immunoprecipitation. Using Transwell experiments, cell invasion and migration were examined. The enzyme-linked immunosorbent assay was utilized to analyze the MMP-2 and MMP-9 secretion levels. RNA pull-down and RNA immunoprecipitation were conducted to detect the binding between ALKBH5 and PLAC8.

**Results:**

In PE tissue and hypoxia-treated HTR-8/SVneo cells, levels of ALKBH5 and PLAC8 were increased, and PLAC8 m6A methylation levels were decreased. There was a positive correlation between PLAC8 and ALKBH5 expression in clinical tissues. In addition, overexpressing PLAC8 promoted HTR-8/SVneo cell migration and invasion, and so as the levels of MMP-2 and MMP-9; while interference with PLAC8 reduced the migration and invasion of hypoxia-treated HTR-8/SVneo cells, and so as the levels of MMP-2 and MMP-9. Moreover, the PLAC8 mRNA’s m6A modification site was GAACU (Position 1449, Site 2). Increased levels of MMP-2 and MMP-9, as well as migration and invasion of HTR-8/SVneo cells exposed to hypoxia, were all facilitated by the m6A Site2 mutation. Furthermore, ALKBH5 could bind to PLAC8, reduce its m6A modification, and promote its expression.

**Conclusion:**

High-expressed ALKBH5 inhibits the m6A level of PLAC8 mRNA and promotes PLAC8 expression, while PLAC8 overexpression can promote hypoxia-induced invasion and migration of HTR-8/Svneo cells, indicating its potential protective function in PE.

## Introduction

Preeclampsia (PE) is one of the leading factors of maternal mortality among diseases threatening the health of mothers and children [1]. PE typically appears after 20 weeks of gestation and is distinguished by high blood pressure, proteinuria, and damage to placental and kidney tissue. Moreover, severe PE will progress to eclampsia, which will cause symptoms such as convulsions and coma [2, 3]. The incidence of PE is 2–8 percent worldwide [4]. The younger the gestational age, the greater the harm. PE is classified as early (occurring before 34 weeks) or late (occurring after 34 weeks) according to the gestational week of onset. Early PE is uncommon, but the disease is severe; perinatal prognosis is poor; severe complications are more likely; not only is the recurrence rate of second pregnancy higher, but the risk of PE in their offspring is higher. According to studies, early PE is primarily a placental disease, whereas late PE is mostly a maternal disease, which may have a variety of pathogenesis, but the precise pathogenesis of PE is yet unknown [5].

At the moment, the most effective treatment for PE is still pregnancy termination and placenta delivery. It is generally accepted that PE develops and progresses due to the abnormal placental trophoblast cell function, which is a pathogenic cause. Placental trophoblast cells have many similarities with tumor cells, but under the regulation of many human factors, the function of trophoblast cells presents strict time and space fine regulation. The inability of trophoblast cells to invade may be related to fetal growth restriction and preeclampsia, and its invasiveness can lead to the occurrence of gestational trophoblastic diseases such as hydatidiform mole and choriocarcinoma [6]. Although PE occurs after 20 weeks of gestation, abnormal placental trophoblast function may begin early in pregnancy and last until the placenta is delivered at the end of the pregnancy. Investigating the factors that influence the biological operation of placental trophoblast cells to serve the purpose of further revealing the pathogenesis of PE.

Placenta-specific 8 (PLAC8) is found in the placenta of mice and is highly conserved [7]. In mouse placentas, the majority of Plac8 mRNA was found in trophoblast giant cells at 6.5 and 8.5 dpc and spongy trophoblast at 10.5 and 18.5 dpc, indicating an important role for Plac8 in placental development. However, the function of PLAC8 in the human placenta has not been fully revealed. One study suggested that PLAC8 is an important biomarker of pregnancy outcomes in mammals [8]. Notably, PLAC8 expression is found to be elevated in PE placenta tissues and hypoxic trophoblast cells, and it is a key factor in placenta development [9, 10]. Considering that the specific upstream regulatory mechanism is unclear, whether PLAC8 can be used as a target to intervene in the progression of PE needs to be further clarified.

N6-methyladenosine (m6A) is a modification common in eukaryotes and plays an important role in development, homeostasis, and disease. As reported, m6A also plays a role in early development and embryogenesis [11]. PLAC8 m6A modification has been reported to be reduced in PE patients' placental tissue [12]. AlkB homologue 5 (ALKBH5) is the second m6A demethylase found in mammals, and it can regulate pre-mRNA processing, mRNA decay, and translation through m6A demethylation [13]. ALKBH5 expression is elevated in PE patients' placental tissues and HTR-8/Svneo cells treated with hypoxia/reoxygenation, while decreasing the m6A modification level of PPARG mRNA by interfering with ALKBH5 expression is helpful to enhance its mRNA stability and protein expression [14]. Our study combined with biogenic analysis to explore the upstream regulatory mechanism of PLAC8 in the progression of PE, that is, whether and how ALKBH5 regulates PLAC8 expression.

In this study, we focused on hypoxic-induced changes in the activity and function of trophoblast cells and clarified the regulatory role of PLAC8 on the activity and function of trophoblast cells. We assumed that ALKBH5 might promote PLAC8 expression by binding to PLAC8, which is related to m6A modification. Furthermore, the up-regulation of PLAC8 promotes placental trophoblast invasion and migration and alleviates PE progression. Exploring the regulatory factors and mechanisms of the activity and dysfunction of placental trophoblastic cells is of great significance to elucidate the pathogenesis of PE and guide its prevention and treatment.

## Materials and methods

### Placental samples

The severe PE samples of 30 pregnant women who underwent cesarean section in the First Affiliated Hospital were selected. The diagnostic criteria for PE were: new diastolic blood pressure ≥ 90 mmHg and/or systolic blood pressure ≥ 140 mmHg after 20 weeks of gestation, with new proteinuria ≥ 0.3 g/24 h, or random urinary protein ( +). Severe PE inclusion criteria: PE with any of the following manifestations, (1) diastolic blood pressure ≥ 110 mmHg, or systolic blood pressure ≥ 160 mmHg; (2) Thrombocytopenia (platelets < 100 × 10^9^/L); (3) Liver function impairment (serum transaminase level more than 2 times the normal value), severe persistent right upper abdomen or upper abdomen pain, which cannot be explained by other diseases; (4) Renal function impairment (creatinine level is greater than 1.1 mg/dL or creatinine concentration is more than 2 times the normal value in the absence of other kidney diseases); (5) Pulmonary edema; (6) New central nervous system abnormalities or visual disturbances. Rule out twin pregnancy, gestational diabetes, kidney disease, chronic hypertension, acute and chronic hepatitis, hyperthyroidism and hypothyroidism. The gestational week and age of pregnant women in the normal control group were matched with those in the preeclampsia group, and no previous history of primary hypertension, diabetes, chronic nephritis, etc. The pregnancy was normal, the fetal development was normal, no pathological chorioamniotic inflammation was found, and no complications or complications were found. The relevant clinical data of patients and controls are shown in Table 1.

**Table 1:** The general information about PE and HC

Groups	HC (n = 30)	PE (n = 30)
Ages (years)	31.2 ± 3.21	29.5 ± 4.69
Gestational weeks	32.3 ± 8.42	32.4 ± 6.68
Systolic pressure (mmHg)	110.7 ± 29.20	150.7 ± 34.14^a^
Diastolic pressure (mmHg)	72.3 ± 19.85	98.1 ± 22.47^a^

^a^*p* < 0.05 vs. HC

Within 5 min after the removal of the placenta, 4 pieces from each placental tissue, about 1 cm^3^ in size, were taken out at points 3, 6, 9, and 12 in maternal surface, at a distance of 2–3 cm from the edge, with the umbilical cord attached as the center (to avoid calcification foci, organization foci, bleeding foci, etc.). Before being frozen in liquid nitrogen, in order to remove any remaining blood, sterile normal saline was used to rinse the collected samples until no blood was present. Following that, samples were transferred to the − 80 ℃ refrigerator for later use. Everyone who took part signed an informed consent form. The study was approved by the Ethics Committee of Scientific Research and Clinical Trials of the First Affiliated Hospital of Zhengzhou University (NO. 2022-KY-0318-002).

### Quantitative real-time PCR (qRT-PCR)

The mRNA levels of ALKBH5 and PLAC8 in placental tissue and trophoblast cells were measured by qRT-PCR. The total mRNA was extracted using Trizol (Beyotime, CHN), followed by reversed to cDNA using an RT-PCR Kit (X–Y Biotechnilogy, Shanghai, CHN). Then, the samples were subjected to the real-time fluorescence quantitative PCR instrument (Applied Biosystems, USA) with SYBR Green PCR Mix (TheroFisher, USA). GAPDH was used as the control [15].

Homo PLAC8: 

F: 5′-GCAACTCTTTGCTGTCCTCA-3′;

 R: 5′-AGGCATGTTTGCATTGACTCAC-3′.

Homo ALKBH5: 

F: 5′-GCTGGTTGCTCCTTTTGAGC-3′;

 R: 5′-CTTGGAAGGACACCAGTCCC-3′.

Homo GAPDH: 

F: 5′-AATGGGCAGCCGTTAGGAAA-3′;

R: 5′-GCGCCCAATACGACCAAATC-3′.

### Western blot analysis

The protein levels of ALKBH5, MMP-2, MMP-9, and PLAC8 in placental tissue and trophoblast cells were measured by western blot. RIPA Lysis Buffer (Beyotime, CHN) containing protease inhibitors was used to cleave the placental tissue and trophoblast cells. The concentration of extracted protein samples were measured using a BCA kit (TheroFisher, USA). The samples were then transferred to a PVDF membrane after going through SDS-PAGE. Soon afterward, the membrane was blocked for 10 min with Protein Free Rapid Blocking Buffer (Epizyme, Shanghai, CHN). Subsequently, the membrane was incubated overnight at 4 °C with Anti-ALKBH5 antibody (ab195377; 1/1000), Plac8 (E1J2Z) Rabbit mAb (#13885; Cell Signaling), Anti-MMP2 antibody (ab92536; 1/2000), Anti-MMP9 antibody (ab76003; 1/2000), and Anti-Tubulin antibody (ab6160; 1/5000). Then, the membrane was incubated at room temperature with the secondary antibody before being immersed in enhanced chemiluminescence and exposed in the dark. The bands were observed with a chemiluminescence imager (TheroFisher, USA) [16].

### Cell treatment

The trophoblast cell line (HTR-8/SVneo cell) was cultured in RPMI 1640 medium supplemented with 1 percent penicillin/streptomycin and 10 percent fetal bovine serum (FBS). Subsequently, hypoxia was applied to the cells. HTR-8/SVneo cells were inoculated in an environment with 93 percent N_2_, 2 percent O_2_, and 5 percent CO_2_ for 24 h. For the Control group, cells were cultured for 24 h with 75 percent N_2_, 20 percent O_2_, and 5 percent CO_2_ [17].

### Methylated RNA immunoprecipitation (MeRIP) assay

MeRIP-qPCR was carried out using the Magna MeRIP m6A Assay (Millipore, Germany). The total mRNA was extracted using Trizol (Beyotime, CHN). Then, the RNA was sheared to a length of about 100 nt using the RNA Fragmentation Buffer. The Magnetic A/G Beads were resuspended and incubated with an anti-m6A antibody (Synaptic Systems, Germany). Afterward, the m6A-Beads complex was incubated with the above-mentioned fragmented RNA for 2 h at 4 ℃. Following elution and purification, the relative PLAC8 m6A levels were measured by qRT-PCR [18].

### Cell transfection

A 6-well plate was seeded with HTR-8/SVneo cells (3 × 10^5^ cells). GenePharma (Shanghai, CHN) designed and synthesized pcDNA-ALKBH5 (or pcDNA-NC), pcDNA-PLAC8 (or pcDNA), or siRNA-PLAC8 (or siRNA-NC) were diluted and then transfected into HTR-8/SVneo cells with Lipofectamine 3000 (Invitrogen, USA). Transfectants in each well were replaced with 2 mL of RPMI 1640 medium containing 10% FBS 8 h after transfection, and the culture was kept going for 16 h before the hypoxia treatment.

### Transwell assay

The migration and invasion of HTR-8/SVneo cells were detected via Transwell assay. The Matrigel was diluted with pre-cooled DMEM medium and added to the center of the chamber to detect invasion. The lower layer of the chamber was filled with medium containing 20% FBS. The cells were resuspended in DMEM medium after trypsin digestion, and the resulting cell suspension was planted at a density of 3 × 10^4^/well in the upper chamber, and incubated in a 37 °C, 5% CO_2_ incubator for 24 h. The transwell chamber was removed, the liquid in the chamber was aspirated, and 4% paraformaldehyde was added to the upper chamber. After 30 min, the cells were washed with PBS and stained for 10 min with the crystal violet solution. Photos were taken under the microscope, 5 fields were randomly selected, and Image J was used to calculate the number of lower transmembrane cells. To detect migration, the protocols were the same as those for the cell invasion assay, with the exception that there was no Matrigel layer.

### Enzyme-linked immunosorbent assay (ELISA)

The secretion levels of MMP-2 and MMP-9 were detected by ELISA. The MMP2 Human ELISA Kit (#KHC3081, Invitrogen) and MMP9 Human ELISA Kit (#BMS2016-2, Invitrogen) were applied following relevant instructions.

### RNA immunoprecipitation (RIP)

Cell lysates containing protease and RNase inhibitors were used to cleave HTR-8/SVneo cells. After resuspending the protein A + G magnetic beads, they were incubated with an anti-ALKBH5 antibody. The complex that formed was then incubated with cell lysate. qRT-PCR was used to determine the relative PLAC8 mRNA levels after elution and purification.

### RNA pull-down assay

The HTR-8/SVneo cells were cleaved with cell lysates containing protease and RNase inhibitors. The biotin-labeled PLAC8 (PLAC8) probe or PLAC8's anti-sense probe (PLAC8-AS) was incubated with magnetic beads before being incubated with cell lysate. After elution and purification, the level of ALKBH5 in the complex was measured using Western blot analysis.

### Statistical analysis

The data was processed using the GraphPad Prism 8 software (San Diego, CA, USA) and displayed as means ± SD. The student’s t-test was used to test differences between two groups, and to test for differences between multiple groups, one-way ANOVA was used, followed by the LSD post hoc test. *P* < 0.05 was deemed significant [19].

## Results

### Expression of PLAC8 and ALKBH5 was up-regulated in the placental tissue of PE patients and hypoxia-induced trophoblast cells.

**Fig. 1:**
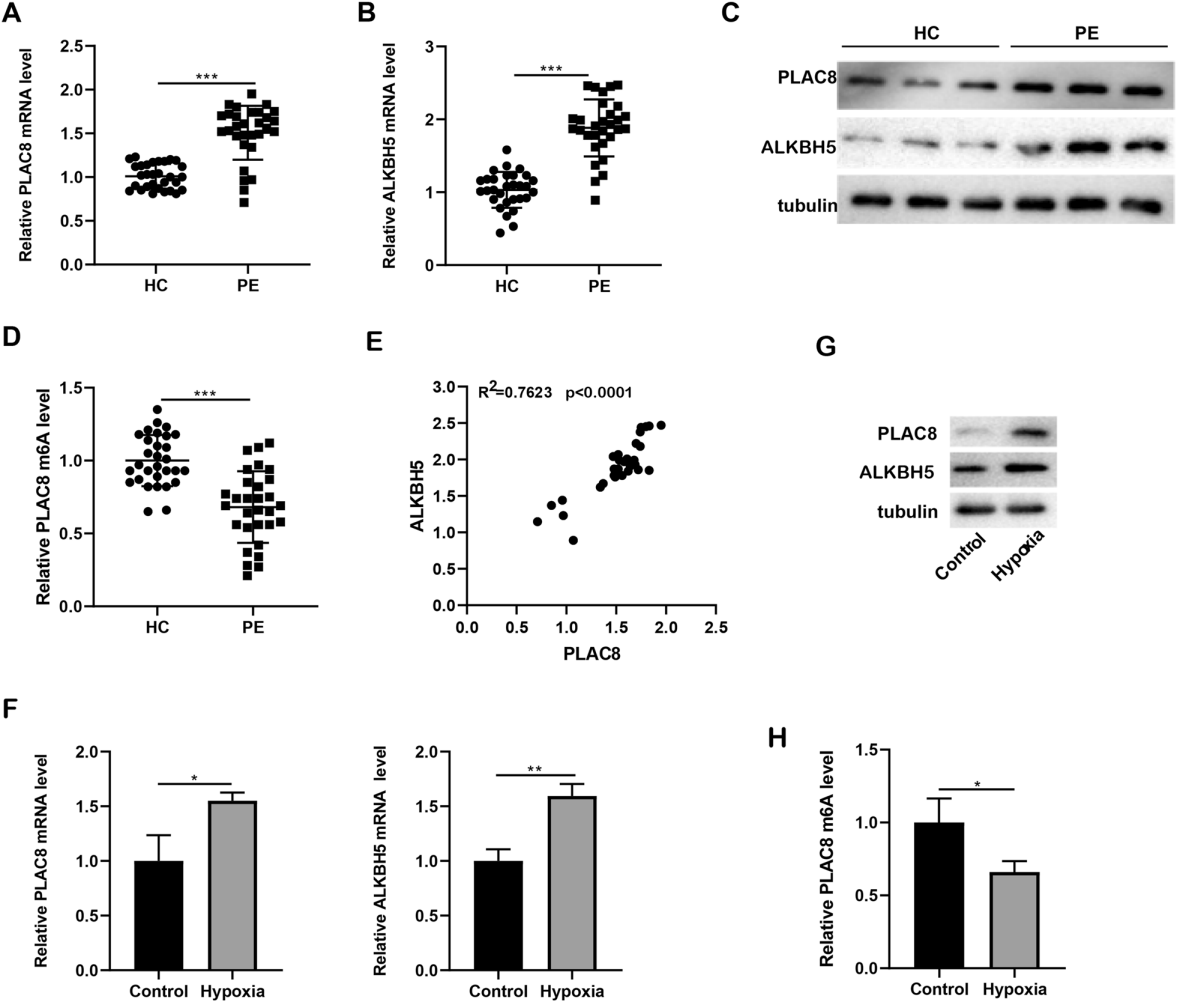
PLAC8 and ALKBH5 expression in placental tissue of PE patients and hypoxia-induced trophoblast cells. The placenta tissue was collected from PE patients (n = 30) and healthy people (HC, n = 30). **A**, **B** The mRNA levels of PLAC8 and ALKBH5 in placenta tissue were detected by qRT-PCR. **C** Protein levels of PLAC8 and ALKBH5 in placenta tissue were detected by western blot. **D** Relative PLAC8 m6A level in clinical samples was detected. **E** The correlation between PLAC8 and ALKBH5 expression in clinical tissues was analyzed. The HTR-8/Svneo cells were induced by hypoxia. **F** The mRNA levels of PLAC8 and ALKBH5 in HTR-8/Svneo cells were detected by qRT-PCR. **G** Protein levels of PLAC8 and ALKBH5 in HTR-8/Svneo cells were detected by western blot. **H** Relative PLAC8 m6A level in hypoxia-induced trophoblast cells was detected. ^*^p < 0.05, ^**^p < 0.01, ^***^p < 0.001

Thirty placental tissue samples were taken from severe PE patients and healthy people, respectively. PLAC8 and ALKBH5 mRNA and protein levels were measured, and the findings demonstrated that PLAC8 and ALKBH5 expression in placental tissue was significantly up-regulated in PE samples as compared to HC samples (*p* < 0.001; Fig. 1A–C). The changes of PLAC8 m6A modification levels in clinical samples were detected, and the results showed a reduction in relative PLAC8 m6A levels in PE samples (n = 30; Fig. 1D). Further research was done on the relationship between PLAC8 and ALKBH5 expression in clinical tissues. We discovered that the connection between PLAC8 expression and ALKBH5 was shown to be favorable (*p* < 0.0001; Fig. 1E), hinting that the increased expression of PLAC8 may be related to the lower amount of m6A modification caused by the increased expression of ALKBH5.

The trophoblast cell line (HTR-8/SVneo cell) was treated with hypoxia, and mRNA and protein levels of PLAC8 and ALKBH5 were examined. The findings demonstrated that after hypoxia treatment, in comparison to the Control group, PLAC8 and ALKBH5 expression in HTR-8/SVneo cells was noticeably increased (*p* < 0.05 or *p* < 0.01; Fig. 1F, G). In HTR-8/SVneo cells treated with hypoxia, relative PLAC8 m6A levels were also decreased (*p* < 0.05; Fig. 1H), which was consistent with the detection in clinical samples.

### Influence of PLAC8 on the migration and invasion of trophoblast cells.

**Fig. 2:**
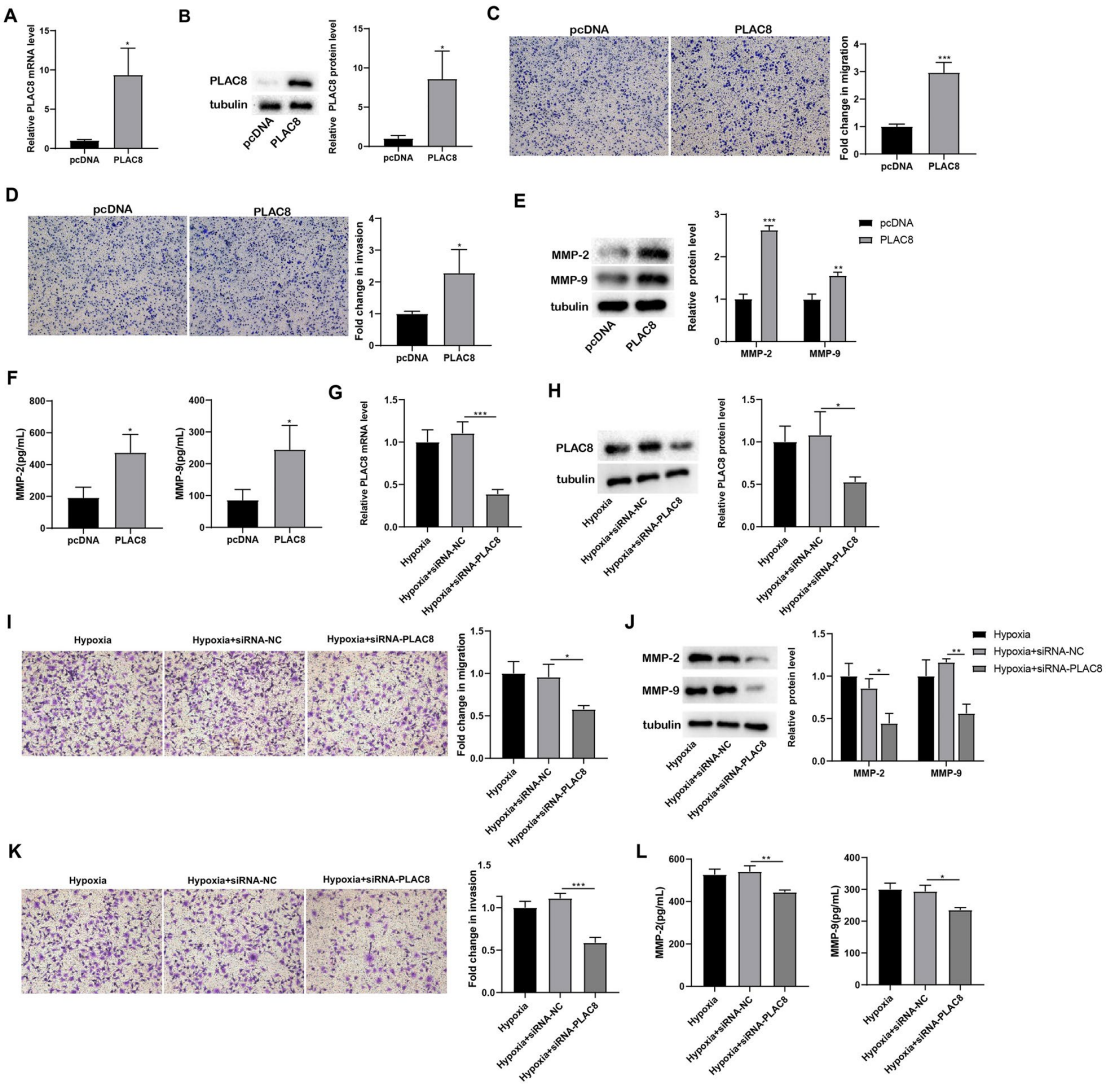
Regulation of PLAC8 on the migration and invasion of trophoblast cells. The HTR-8/SVneo cells were transfected with PLAC8 (or pcDNA). N = 3. **A** The mRNA levels of PLAC8 in HTR-8/Svneo cells were detected by qRT-PCR. **B** Protein levels of PLAC8 in HTR-8/Svneo cells. **C**, **D** Cell migration and invasion were detected by the Transwell assay. **E** Protein levels of MMP-2 and MMP-9 in HTR-8/Svneo cells were detected by western blot. **F** The secretion levels of MMP-2 and MMP-9 in HTR-8/Svneo cells were detected by ELISA. The HTR-8/SVneo cells were transfected with siRNA-PLAC8 (or siRNA-NC) before being treated with hypoxia. **G** The mRNA levels of PLAC8 in HTR-8/Svneo cells were detected by qRT-PCR. **H** Protein levels of PLAC8 in HTR-8/Svneo cells. **I**, **K** Cell migration and invasion were detected by the Transwell assay. **J** Protein levels of MMP-2 and MMP-9 in HTR-8/Svneo cells were detected by western blot. **L** The secretion levels of MMP-2 and MMP-9 in HTR-8/Svneo cells were detected by ELISA. ^*^p < 0.05, ^**^p < 0.01, ^***^p < 0.001

The HTR-8/SVneo cells were transfected with PLAC8 to overexpress PLAC8 expression (*p* < 0.05; Fig. 2A, B). Besides, when compared to the pcDNA group, the migration and invasion in the PLAC8 group increased significantly (*p* < 0.05 or *p* < 0.001; Fig. 2C, D). Western blot was applied to detect the expression levels of MMP-2 and MMP-9, and the findings demonstrated that overexpressing PLAC8 dramatically increased the expression of MMP-2 and MMP-9 (*p* < 0.01 or *p* < 0.001; Fig. 2E). ELISA was performed to examine the secretion levels of MMP-2 and MMP-9, and response to PLAC8 overexpression, MMP-2 and MMP-9 secretion increased (*p* < 0.05; Fig. 2F).

The HTR-8/SVneo cells were then transfected with siRNA-PLAC8 to inhibit PLAC8 expression, followed by hypoxia induction (*p* < 0.05 or *p* < 0.001; Fig. 2G, H). Besides, when compared to the siRNA-NC group, the migration and invasion in the Hypoxia + siRNA-PLAC8 group had a definite declining tendency (*p* < 0.05 or *p* < 0.001; Fig. 2I, K). Western blot was applied to detect the expression levels of MMP-2 and MMP-9, and the findings demonstrated that PLAC8 interference remarkably decreased the expression of MMP-2 and MMP-9 (Fig. 2J). ELISA was performed to examine the secretion levels of MMP-2 and MMP-9, and as a result of siRNA-PLAC8 transfection, MMP-2 and MMP-9 secretion was found to be reduced (*p* < 0.05 or *p* < 0.01; Fig. 2L).

### Expression and functional regulation of PLAC8 mediated by m6A modification.

**Fig. 3:**
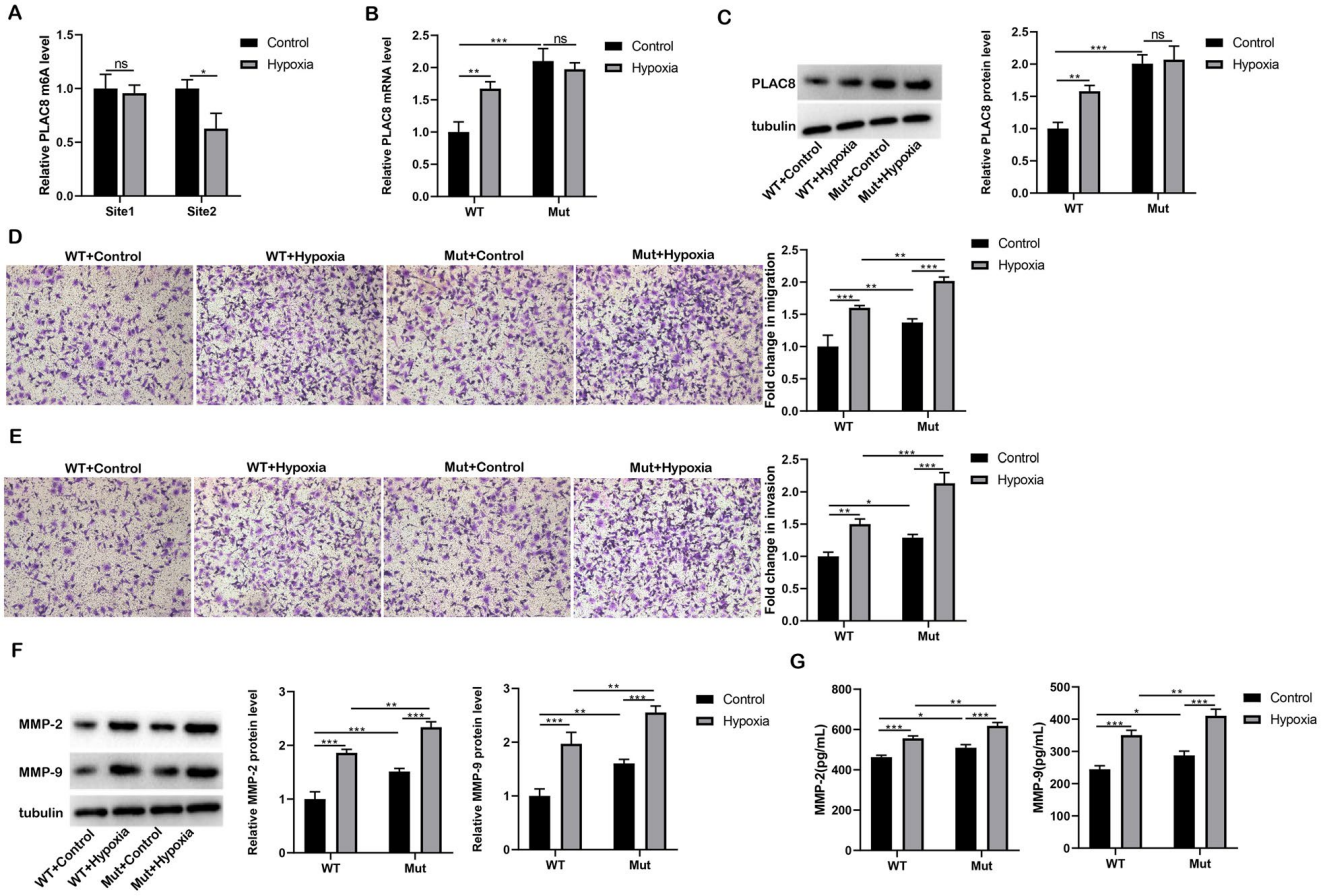
Expression and functional regulation of PLAC8 mediated by m6A modification. **A** The m6A levels of GGACU (Position 405) (Site1) and GAACU (Position 1449) (Site2) in HTR-8/SVneo cells treated with hypoxia were detected. **B**, **C** PLAC8 expression was detected by qRT-PCR and Western blot. **D**, **E** Transwell was performed to detect migration and invasion. **F** Protein levels of MMP-2 and MMP-9 in HTR-8/Svneo cells were detected by western blot. **G** The secretion levels of MMP-2 and MMP-9 in HTR-8/Svneo cells were detected by ELISA. ^ns^ *p* > 0.05, ^*^p < 0.05, ^**^p < 0.01, ^***^p < 0.001

We further explored the possible mechanism of PLAC8 abnormal expression in trophoblastic cells under hypoxia. The potential m6A modification sites GGACU (Position 405, Site1) and GAACU (Position 1449, Site2) in the PLAC8 mRNA sequence were found to have high credible values by SRAMP analysis. The changes of m6A levels in the above two regions in hypoxia-treated HTR-8/SVneo cells were detected. Figure 3A gave evidence that the enrichment level of GAACU (Position 1449, Site2) was significantly reduced (*p* < 0.05) after hypoxia treatment, proposing that Site2 was the m6A modification site of PLAC8 mRNA. A mutant expression vector (A → G) of PLAC8 m6A modification site GAACU (Position 1449) was constructed, namely pcDNA-PLAC8-MUT. The pcDNA-PLAC8-WT or pcDNA-PLAC8-MUT was transfected into HTR-8/SVneo cells, followed by hypoxia treatment. Western blot and qRT-PCR analysis of PLAC8 confirmed that hypoxia had no significant effect (*p* > 0.05) on PLAC8 expression in the pcDNA-PLAC8-MUT group (Fig. 3B, C). The Transwell assay revealed that either hypoxia treatment alone or m6A Site2 mutation could promote cell migration (*p* < 0.001 or *p* < 0.01) and invasion (*p* < 0.01 or *p* < 0.05), and m6A Site2 mutation further promoted hypoxia-induced cell migration and invasion (*p* < 0.01 or *p* < 0.001; Fig. 3D, E). As for the levels of MMP-2 and MMP-9, it was found that hypoxia promoted the MMP-2 and MMP-9 expression and secretion (*p* < 0.001), and MMP-2 and MMP-9 production and secretion were further aided by m6A Site2 mutation (*p* < 0.01; Fig. 3F, G).

### ALKBH5 reduces PLAC8 m6A modification and promotes its expression

**Fig. 4:**
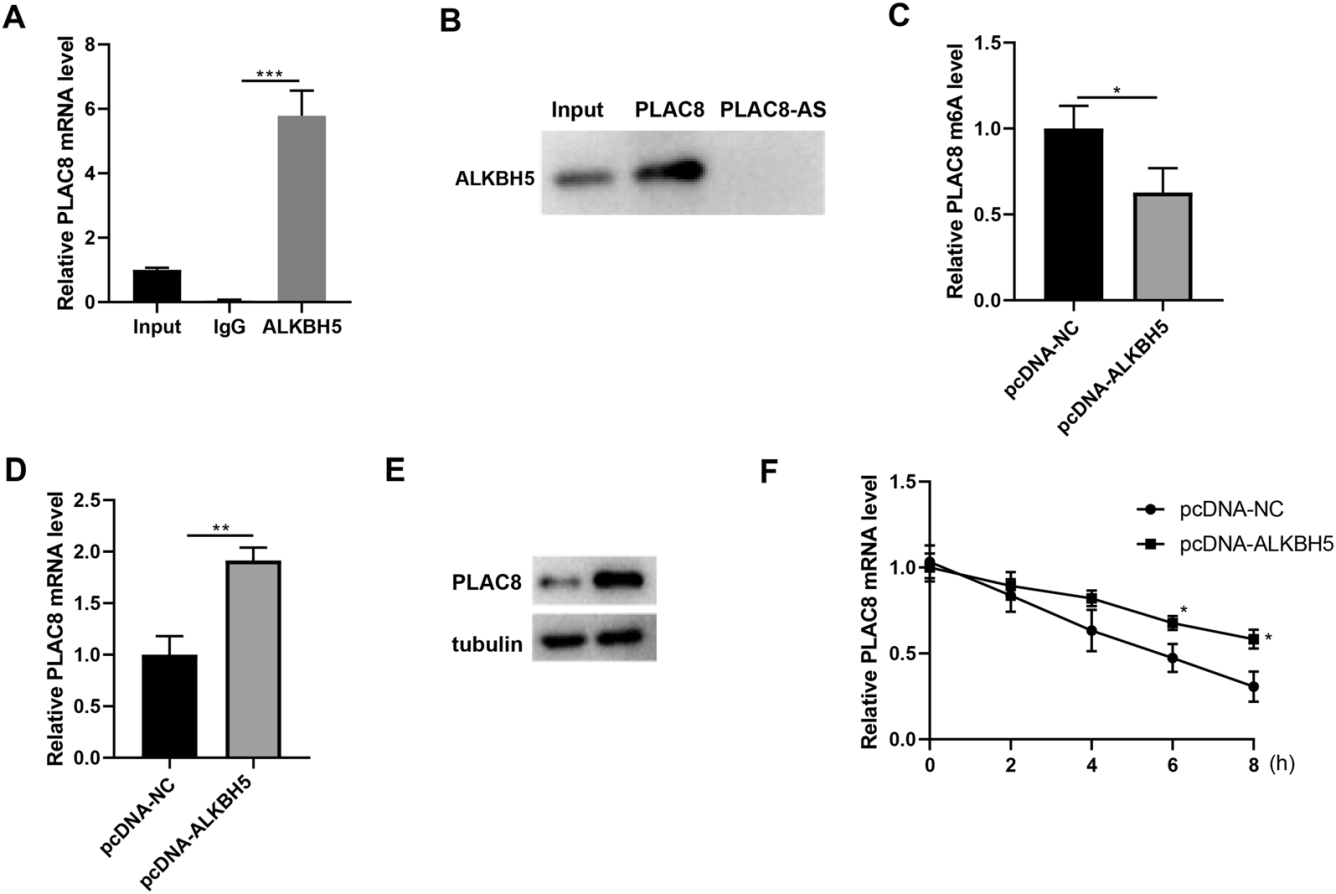
Effect of ALKBH5 on PLAC8 m6A modification. **A** RIP assay was performed in HTR-8/SVneo cells. **B** RNA-pull down assay was performed in HTR-8/SVneo cells. The HTR-8/SVneo cells were transfected with pcDNA-ALKBH5 (or pcDNA-NC). N = 3. **C** The MeRIP assay was used to detect the PLAC8 m6A level. **D**, **E** PLAC8 expression was detected by qRT-PCR and Western blot. **F** The mRNA levels of PLAC8 in HTR-8/Svneo cells were detected by qRT-PCR. ^*^p < 0.05, ^**^p < 0.01, ^***^p < 0.001

According to Fig. 1’s findings, in both the PE placental tissue and the trophoblast cell injury models, the ALKBH5 level was significantly elevated, implying that ALKBH5 may be involved in the progression of PE. Given that PLAC8 expression is positively linked to ALKBH5 expression in the placenta of PE patients, and that ALKBH5 protein may interact with PLAC8 mRNA, it is possible that decreased PLAC8 m6A modification is associated with increased expression of ALKBH5. In HTR-8/Svneo cells, we performed a RIP assay, and the PLAC8 mRNA was found to be enriched in ALKBH5 antibody precipitation (Fig. 4A). An endogenous RNA-pull-down assay revealed that the PLAC8 RNA probe was found to be capable of pulling down ALKBH5 protein via Western blot to examine the pull-down product of PLAC8 mRNA (Fig. 4B). Secondly, the regulation of PLAC8 expression by ALKBH5 was clarified. HTR-8/Svneo cells were transfected with pcDNA-ALKBH5. MeRIP assay was performed to detect the m6A methylation level of PLAC8 mRNA, which was found to be decreased after ALKBH5 overexpression (pcDNA-ALKBH5; Fig. 4C). PLAC8 mRNA and protein expression were detected by qRT-PCR and Western blot. The outcomes demonstrated that ALKBH5 positively regulated PLAC8 expression (*p* < 0.01; Fig. 4D, E). Subsequently, HTR-8/Svneo cells were transfected with pcDNA-ALKBH5 and then treated with an RNA synthesis inhibitor-actinomycin D (ActD; 5 μg/mL), PLAC8 mRNA levels were examined through qRT-PCR after 0, 2, 4, 6, and 8 h. From 6 h on, relative PLAC8 mRNA expression in the pcDNA-ALKBH5 group was dramatically higher than in the pcDNA-NC group (*p* < 0.05; Fig. 4F), indicating that ALKBH5 could inhibit PLAC8 mRNA degradation.

## Discussion

PE is a multifactorial, multi-pathway, multi-presenting disease with continuity, heterogeneity, and instability [20]. Fetal intrauterine growth restriction, maternal death, premature birth, and stillbirth are all painful experiences for the patient's family [21]. Women with PE are more likely than healthy women to develop atherosclerosis, stroke, and other vascular diseases [22], indicating that women’s health and fetal development are both significantly impacted by PE. Given the uniqueness of pregnancy, clinical medication is extremely cautious. Pregnancy termination is the only viable treatment at this time because there is no appropriate medicine for intervention [23]. PE poses a major health risk to both mother and child's health, but its pathogenesis is still not entirely known. ALKBH5 and PLAC8 were discovered to be up-regulated at the mRNA and protein levels in placental tissues from PE patients as well as hypoxia-induced HTR-8/Svneo cells in the study. Additionally, interference with PLAC8 lessened the migration and invasion of HTR-8/Svneo cells brought on by hypoxia. Furthermore, the m6A level and mRNA degradation of PLAC8 may be impacted by an interaction with ALKBH5.

The placenta, as an auxiliary organ of the fetus, serves as an essential link between the mother and the fetus [24]. Many perinatal disorders, including PE, are caused by abnormalities in placental function. According to clinical trials, high blood pressure and proteinuria during pregnancy disappear shortly after the placenta is delivered [25]. As a consequence, many scientists deem that PE is a placental-derived disorder, with trophoblastic dysfunction eventually leading to placental shallow implantation [26, 27]. The placental trophoblast cells are the executing cells of placental invasion and uterine vascular recasting. Placental development disorder is caused by abnormal activity and function of placental trophoblast cells. In this current research, we probed into the alterations in migration and invasion using the placental tissue samples and hypoxia-induced trophoblast cell line HTR-8/Svneo that had been collected as subjects.

PLAC8 has been shown in fore research to be crucial in tumor progression by regulating cell apoptosis, differentiation, and proliferation [28]. Besides, in various species, PLAC8 is required for embryonic development, and at all times during the early stages of human embryonic development, the PLAC8 protein is expressed [29]. PLAC8 also regulates trophoblast cell autophagy, and its overexpression can boost trophoblast cell viability and proliferation while protecting cells from etoposide toxicity [30]. PLAC8 was discovered to be highly expressed in PE tissues and hypoxia-induced HTR-8/Svneo cells in our study, which is consistent with Chang et al.’s discovery [9]. In addition, overexpressing PLAC8 promoted HTR-8/SVneo cell migration and invasion, as were the levels of MMP-2 and MMP-9; while PLAC8 inhibition suppressed the migration and invasion of HTR-8/Svneo cells under hypoxia, as were the levels of MMP-2 and MMP-9.

Epigenetics has been implicated in the development of PE [31, 32]. *Ohgane *et al. discovered that the DNA methylation state influenced gestation trophoblast differentiation [33]. M6A methylation is the most common mRNA modification in the majority of eukaryotes and concerns virtually every phase of the RNA life cycle, including RNA transcription, translation, and degradation. It has been claimed that the m6A modification level of PLAC8 mRNA in placental tissues of PE patients was significantly altered, that is, a decrease in the level of m6A modification coincides with an increase in PLAC8 mRNA expression [12]. Consistent with this, in our study, placental tissue and hypoxic-induced trophoblast cells showed increased expression levels of PLAC8 mRNA and decreased m6A modification levels of PLAC8 mRNA. In addition, in the placenta of PE patients, the overall amount of m6A modification has been reported to be increased, as well as the expression of METTL3 and METTL14 (m6A methylases) [34, 35], indicating that the up-regulation of some m6A demethylases may be responsible for the PLAC8 mRNA's lower level of m6A modification. Similarly, we discovered that in PE tissues and hypoxia-induced HTR-8/Svneo cells, the decreased m6A modification of PLAC8 mRNA was associated with the elevation of the m6A demethylase ALKBH5.

ALKBH5 is an m6A demethylase that regulates mRNA degradation and may contribute to the development of numerous illnesses [36, 37]. ALKBH5 inhibition has been shown to promote HTR-8/Svneo cell migration by reducing m6A-modified PPARG mRNA [14]. The most recent evidence also demonstrated that specific knockdown of ALKBH5 expression in mouse placental trophoblast inhibited trophoblast cell invasion, making for fetal abortion; additional studies revealed that ALKBH5 transferred from the nucleus to the cytoplasm after hypoxia treatment, causing m6A demethylation of some target mRNAs and subsequently enhancing translation expression of corresponding mRNAs [38]. Consistent with the above studies, our study showed that ALKBH5 expression was elevated in the placental tissue of PE patients and the trophoblast cell damage model. Furthermore, overexpressing ALKBH5 promoted PLAC8 expression while decreasing its m6A methylation.

According to online analysis software (http://www.rnainter.org/PRIdictor/) [39], PLAC8 mRNA may interact with ALKBH5. Our data support the interaction of ALKBH5 protein and PLAC8 mRNA for the first time. Moreover, overexpressing ALKBH5 reduced the PLAC8 mRNA’s m6A methylation and prevented its degradation. We further used the online software SRAMP (http://www.cuilab.cn/sramp/) [40] to forecast the m6A methylation site on PLAC8 mRNA. The findings revealed that the m6A motif of PLAC8 mRNA could be found at positions 405 (Site 1) and 1449 (Site 2). By detecting the changes of m6A levels in the above two regions in HTR-8/SVneo cells treated with hypoxia, the results demonstrated that position 1449 (Site2) was the PLAC8 mRNA m6A modification site. We then constructed the mutant expression vector of position 1449 (Site2)-pcDNA-PLAC8-MUT and it was found that hypoxia promoted cell migration and invasion, and the levels of MMP-2 and MMP-9, while m6A Site2 mutation further aggravated the above effects.

In summary, our findings suggest that elevated PLAC8 expression in PE patients or trophoblast cell injury models is regulated by ALKBH5-mediated m6A demethylation modification. Hypoxia-induced increase of ALKBH5 expression in HTR-8/Svneo cells reduces the m6A modification level of PLAC8 mRNA, increases PLAC8 expression level, and the up-regulation of PLAC8 promotes hypoxia-induced invasion and migration of trophoblast cells. Clarifying the regulatory effect of PLAC8 on trophoblast migration and invasion under hypoxia and its upstream regulatory mechanism will not only help to understand the mechanism underlying PE but also have positive significance for the prevention and treatment of PE. Exploring the key regulatory factors of placental trophoblast activity and dysfunction and screening out potential therapeutic targets will help improve perinatal outcomes.

## Data Availability

The datasets used and/or analyzed during the current study are available from the corresponding author on reasonable request.
